# Efficacy and influencing factors of acupuncture in major depressive disorder: a systematic review and exploratory network meta-analysis

**DOI:** 10.1017/S1092852926100868

**Published:** 2026-03-02

**Authors:** Hui Zhao, Yuchen Zhang, Huan Cui, Lingxiang Tang, Yuan Gao, Cheng Tang, Weidong Shen

**Affiliations:** 1 Department of Acupuncture, Shuguang Hospital Affiliated to Shanghai University of Traditional Chinese Medicine, Shanghai, China; 2 Department of Traditional Chinese Massage, Shuguang Hospital Affiliated to Shanghai University of Traditional Chinese Medicine, Shanghai, China

**Keywords:** Acupuncture, major depressive disorder, efficacy, influencing factors, systematic review, meta-analysis

## Abstract

**Background:**

Acupuncture is a clinically recognized treatment for major depressive disorder (MDD), but the associations of efficacy with dosage, treatment course, frequency, acupuncture modality, needle retention time, and manipulation remain unclear. This study evaluated the efficacy and safety of acupuncture for MDD and explored potential moderating factors.

**Methods:**

Randomized controlled trials of acupuncture for MDD were searched in CNKI, VIP Database, Wanfang Data, SinoMed, PubMed, Embase, Web of Science, and the Cochrane Library from inception to May 2025. Risk of bias was assessed using RoB 2, and certainty of evidence using GRADE. Data were analyzed in Stata 18.0.

**Results:**

36 trials involving 3843 participants were included. Compared with sham/placebo acupuncture, acupuncture showed greater antidepressant effects (SMD −1.12, 95% CI −1.57 to −0.67, P < 0.01). Very low-quality evidence suggested similar efficacy between acupuncture and antidepressants. Electroacupuncture was superior to manual acupuncture (SMD −0.24, 95% CI −0.42 to −0.07, P < 0.01). High- and moderate-dose acupuncture were more effective than low-dose regimens, and meta-regression suggested a linear dose-response relationship, with 30 sessions as the optimal dosage. Better outcomes were associated with treatment course >6 wk, 3 times weekly, needle retention time of 20–30 minutes, and electroacupuncture. No significant difference was found between needle manipulation and non-manipulation.

**Conclusions:**

Acupuncture significantly alleviates depressive symptoms in MDD. Efficacy appears to be influenced by dosage, acupuncture modality, treatment course, frequency, and needle retention time, with 30 sessions, treatment course > 6 wk, 3 sessions weekly, 20–30 minutes retention, and electroacupuncture showing the most favorable outcomes.

## Introduction

Depression, also referred to major depressive disorder (MDD), is one of the most prevalent and burdensome psychiatric disorders. MDD is highly correlated with stress, featuring core symptoms including persistent low mood, anhedonia, appetite loss, sleep disturbances, and diminished self-worth.^1^ In severe cases, psychotic features such as hallucinations and delusions may manifest. Epidemiological study^2^ reports MDD’s lifetime prevalence reaches 19%, affecting over 350 million individuals worldwide. Its high disability burden has positioned MDD as a paramount global public health challenge.^3^ According to World Health Organization research data, MDD ranked second in the Global Burden of Disease in 2020 and is expected to rise to first place by 2030, posing persistent and severe encumbrance to socioeconomic systems and healthcare infrastructures worldwide.^4^

Over the past two decades, ketamine and esketamine have been established as rapid-acting antidepressants for MDD. However, prolonged administration may be associated with neuroadaptive changes and potential cognitive impairment, with unassured long-term efficacy maintenance and quality-of-life outcomes.^5^ First-line selective serotonin reuptake inhibitors (SSRIs) similarly present major clinical challenges, including insufficient remission rates, high relapse rates upon discontinuation, poor patient adherence, and delayed onset of action.^6^^–^^8^ Research^9^ has shown that 70% of patients exhibit inadequate response to initial antidepressant therapy, while 30–50% face relapse risk post-treatment cessation. Chronic antidepressant use may induce pharmacological tolerance, dependence, and in some cases, organ toxicity.^10^ Therefore, there is an urgent need for novel, ecologically benign therapeutic strategies to address these critical limitations.

Acupuncture represents a traditional intervention with demonstrated efficacy and favorable safety profile, primarily exerting regulatory effects through exogenous stimulation. While the underlying mechanisms of acupuncture for MDD remain incompletely elucidated, contemporary researches^11^^–^^13^ have characterized its antidepressant actions from multiple perspectives, namely neuroplasticity, hypothalamic–pituitary–adrenal axis homeostasis, immune activation, neuroinflammation, and the microbiota–gut–brain axis, along with epigenetic regulation, neural circuit functional remodeling, glial–neuronal interactions, and neurotransmitter system rebalancing. In terms of clinical efficacy validation, although Cochrane systematic reviews provide evidence supporting its efficacy in MDD treatment,^14^^–^^16^ significant variability exists across studies. Some report greater clinical remission rates for acupuncture monotherapy or adjunctive use compared to conventional SSRIs, while others show only marginal superiority over placebo.^17^ This heterogeneity may be attributed to differences in acupuncture modalities, dosage, and manipulation techniques. Dose–effect linear relationships were found between acupuncture sessions and Hamilton Depression Rating Scale (HAMD) score reduction.^18^ A network meta-analysis (NMA) identified electroacupuncture plus SSRIs as producing the largest effect sizes.^19^ However, only a limited number of studies have explored the optimal acupuncture dosage and the efficacy of different acupuncture modalities, and there is a lack of comprehensive research to elucidate the potential factors influencing the antidepressant effects of acupuncture. To fill the theoretical gap, the present research aimed to evaluate the acupuncture efficacy and safety in MDD, specifically examining the dose–effect, treatment course–effect, treatment frequency–effect, acupuncture modalities–effect relationships, as well as whether the antidepressant efficacy of acupuncture varies with needle retention time and needling manipulation.

## Methods

As a systematic review and meta-analysis, this study strictly adheres to the Preferred Reporting Items for Systematic Reviewsand Meta-Analyses (PRISMA) statement guidelines,^20^ and has been prospectively registered in the International Prospective Register of Systematic Reviews (PROSPERO; registration number: CRD420251043344).

### Literature search

Two investigators (H.Z. and Y.Z.) independently conducted comprehensive literature searches across the following databases from inception to May 2025: CNKI, Wanfang Database, VIP Journal Database, SinoMed, PubMed, Embase, Web of Science, and Cochrane Library (see Supplementary File 1). The search strategy incorporated both database-specific Medical Subject Headings terms (MeSH terms) and free-text keywords. No language restrictions were applied during literature screening. A sample search strategy for the Cochrane Library database is presented below:

**Table:** 

Search strategy for Cochrane library database.	
**#1**	Depression [all text]
**#2**	Depressive Disorder, Major [all text]
**#3**	(“depression”): ti.ab.kw OR (“depressive disorder*”): ti.ab.kw OR (“major depressive disorder”): ti.ab.kw OR (Depressive Symptom*): ti.ab.kw OR (Emotional Depression): ti.ab.kw
**#4**	#1 OR #2 OR #3
**#5**	Acupuncture [all text]
**#6**	(“acupuncture”): ti.ab.kw OR (acupuncture therapy): ti.ab.kw OR (“acupoint*”): ti.ab.kw OR (electroacupuncture*): ti.ab.kw OR (auriculoacupuncture): ti.ab.kw
**#7**	#5 OR #6
**#8**	Randomized Controlled Trials as Topic [all text]
**#9**	(Randomized Controlled Trials): ti.ab.kw OR (trial): ti.ab.kw
**#10**	#8 OR #9
**#11**	#4 AND #7 AND #10

### Study selection

Literature screening was independently performed by 2 reviewers (H.Z. and C.T.). Any discrepancies were resolved through adjudication by a third reviewer (W.S.).

### Inclusion criteria

- Study design: Randomized controlled trials;
- Population: Patients aged 18 years or older, meeting depression diagnostic criteria per the Chinese Classification of Mental Disorders (Third Edition), International Classification of Diseases, or Diagnostic and Statistical Manual of Mental Disorders (DSM-III through DSM-5 TR editions);
- Interventions: The experimental group received any form of acupuncture therapy, while control interventions comprised sham acupuncture (SA), placebo acupuncture, antidepressants, or blank control.

### Exclusion criteria

- Diagnosis of post-stroke depression, postpartum depression, and pregnancy depression.
- Perimenopausal depression and secondary depression due to other medical conditions.
- Per-group sample size ≤15.
- No information was reported regarding randomization methods, ethical approval, or clinical trial registration.

Our study is a paired and exploratory NMA comparing RCTs of acupuncture versus SA, placebo acupuncture, antidepressants, and blank control in patients with MDD. The blank control was defined as acupuncture combined with one or more baseline therapies compared to those same therapies alone (eg, acupuncture plus antidepressants versus antidepressant monotherapy). For 3-arm trials, studies were included if at least 2 arms met the eligibility criteria.

### Outcome analyses

The primary outcome was depressive symptomatology, measured using standardized depression rating scales. When multiple depression metrics were reported in a single study, we prioritized them in the following hierarchical order: (1) HAMD, (2) Montgomery-Asberg Depression Rating Scale (MADRS), and (3) Self-Rating Depression Scale (SDS), and (4) Patient Health Questionnaire-9 (PHQ-9). The secondary outcome was adverse events (AEs) during the trial period, systematically reported regardless of their potential association with acupuncture treatment.

### Data extraction

Three investigators (H.Z., Y.Z., C.T.) independently extracted data, including participant numbers, age, country, acupuncture intervention protocol, treatment duration or frequency, MDD diagnostic criteria, acupoint selection, needle manipulation details, outcome measures (HAMD, MADRS, SDS, PHQ-9 scores), and adverse event incidence. Two senior researchers (Y.G., W.S.) cross-verified all entries for accuracy. For quantitative outcomes, we extracted the mean and standard deviation before and after treatment. When essential data were missing, the corresponding authors of the certain researches were contacted for supplementation.

### Risk-of-bias assessment and certainty of the evidence

The revised Cochrane Risk of Bias tool 2 (RoB 2) was employed to assess the risk of bias across 7 domains in RCTs: randomization process, deviations from intended interventions, missing outcome data, measurement of the outcome, selection of the reported result, and overall bias.^21^ Dual independent evaluations were conducted by reviewers H.Z. and Y.Z., with any discrepancies resolved through consensus discussion involving a third reviewer (H.C.). For evidence synthesis, we employed the Grading of Recommendations, Assessment, Development, and Evaluation (GRADE) methodology^22^ to classify the quality of evidence into 4 levels: high, moderate, low, or very low.

### Data synthesis and analysis

All retrieved results were imported into NoteExpress v4.1.0.10121 for management. In cases where 3-arm studies were included, the sample size of the common control group was divided by 2 to enable 2 independent comparisons.^23^ The primary outcome of this meta-analysis was the difference in depression scale scores between patients receiving acupuncture therapy and those receiving other treatment modalities. Treatment effects were harmonized by converting scores to a common reference scale within each domain. Specifically, depression outcomes were standardized to the 0–56-point HAMD-17. Due to variations in baseline characteristics across studies, we extracted outcome measures before and after the intervention and synthesized effect sizes using changes in mean values and standard deviations (SDs). The SDs of these changes were calculated using the formula recommended in the Cochrane Handbook: SD_change_ = square root [SD^2^_baseline_ + SD^2^_final_ − (2 × Corr×SD_baseline_ × SD_final_)] Corr = 0.4.^24^

Heterogeneity among the included studies was quantified using the I^2^ with P-values. A random-effects model was employed when significant heterogeneity was present (I^2^ > 50% and *p*-value < 0.1); otherwise, a fixed-effects model was used.^25^ Due to variations in outcome measurement scales, treatment effects were expressed as standardized mean differences (SMDs) with 95% confidence intervals (CIs), while dichotomous variables were assessed via risk ratios (RRs).

Subgroup analyses were performed based on acupuncture dosage, treatment course (using a 6-wk cutoff), treatment frequency (3 times a week and 5 times a week), needle manipulation (whether performed or not), and needle retention time (stratified into 20–30 min and 40–50 min groups). Additionally, funnel plots were used to assess publication bias in subgroups with more than 10 studies (see Supplementary File 2). All statistical analyses were conducted using Stata version 18.0.

### The dose–effect analysis

To precisely determine the dose–effect relationship of acupuncture, we restricted the experimental group to acupuncture interventions only, without imposing limitations on comparator treatments. Our primary dose–effect analysis defined treatment dosage as the total number of treatment sessions. Studies were ranked by total dosage from low to high, and subsequently stratified into high (last third of the total), medium (two-thirds of the total), and low-dose subgroups (first third of the total). Besides, a meta-regression analysis of the dose–response relationship was conducted using the robust error meta-regression (REMR) method^26^ to investigate the relationship between the number of acupuncture sessions and the reduction in HAMD scores. Each acupuncture-related experimental group in the included studies was considered a cluster, and a meta-regression model was fitted to the data using the “one-stage” framework of the REMR method. During the pooling process, each specific dose–effect relationship was weighted to balance the heteroscedasticity in the REMR model and ensure the unbiasedness of parameter estimation. The data analysis was performed using the REMR module in Stata 18.0 software. To strike a balance between curve smoothness and accuracy, 3 knots were set (10, 25, 40) to fit the nonlinear relationship.

### The course–effect analysis

When examining the course–effect relationship of acupuncture, potential confounding factors were controlled by restricting the treatment frequency to 2–3 sessions/wk. Studies were stratified into 2 subgroups using a 6-wk cutoff (≤6 wk versus >6 wk), and separate analyses were conducted to evaluate the course–effect relationship for both electroacupuncture (EA) and manual acupuncture (MA).

### The frequency–effect analysis

To address the potential confounding effect of total treatment dose, a predefined subgroup analysis was conducted by restricting the analysis to studies employing a total of 24–30 treatment sessions. To measure the frequency–effect relationship, treatment frequency was divided into 2 groups: 3 times a week and 5 times a week.

### Exploratory network meta-analysis

We conducted an NMA to compare the therapeutic efficacy of EA, MA, auricular acupuncture (AA), and SA. Local inconsistency was assessed using the node-splitting approach to examine congruence between direct and indirect effect estimates. A *p*-value > 0.05 indicated no significant discrepancy between direct and indirect comparisons, warranting application of the consistency model; otherwise, an inconsistency model was employed. For treatment hierarchy assessment, we generated rankograms and calculated the surface under the cumulative ranking curve (SUCRA) probabilities for all interventions with respect to primary outcomes.

## Results

### Search and selection

**Figure 1.**
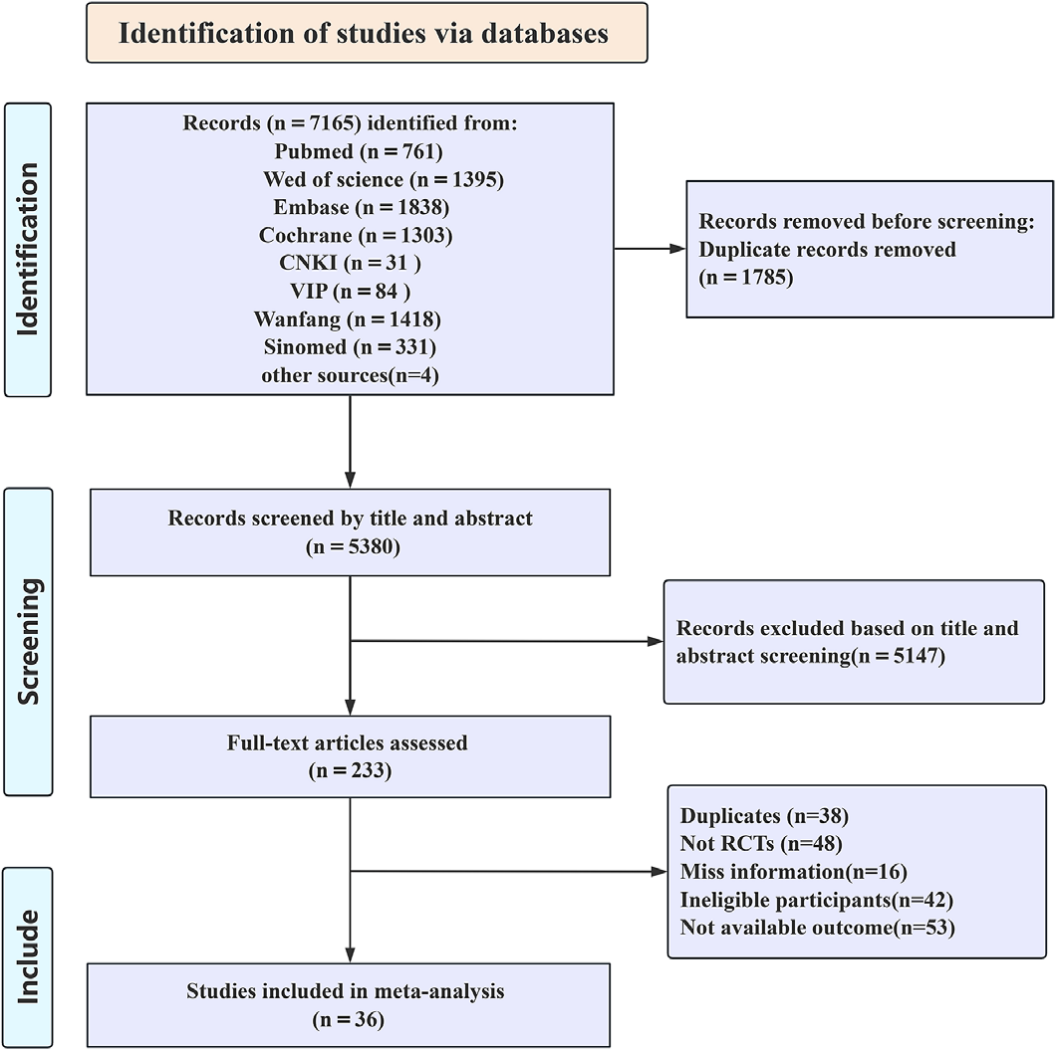
Selection of studies through review.

The initial literature search yielded 7165 references. After removing 1785 duplicates, 5380 records proceeded to screening. Based on title and abstract review, 5147 articles were excluded, leaving 223 potentially eligible studies. Following full-text assessment, 36 RCTs were ultimately included. The detailed selection process is illustrated in Figure 1. Among the 36 RCTs published in 2011–2025, 5 studies compared acupuncture versus antidepressants, 13 studies evaluated acupuncture against SA/placebo acupuncture, 21 studies contrasted acupuncture with no-treatment controls, and 5 studies directly compared EA and MA. Regarding safety, 17 studies reported AEs (eg, dizziness, nausea/vomiting, sleep disturbances). The detailed characteristics of included studies are presented in Supplementary File 3.

### Risk-of-bias assessment of depression outcomes

**Figure 2.**
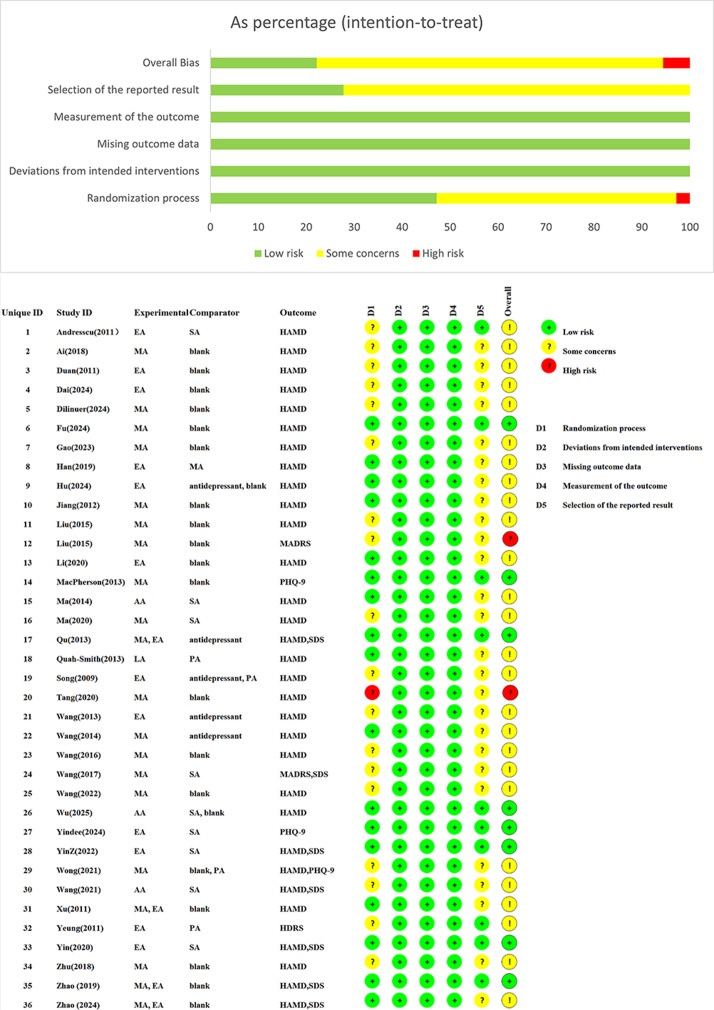
Detailed risk-of-bias assessment. AA, auricular acupuncture; EA, electroacupuncture; MA, manual acupuncture; MADRS, Montgomery-Asberg Depression Rating Scale; PA, placebo acupuncture; PHQ-9, Patient Health Questionnaire-9; SA, sham acupuncture; SDS, Self-rating Depression Scale.

Among the 36 included studies, 2 demonstrated a high risk of bias, 26 were identified as having some concerns, and 8 showed a low risk. The most prevalent bias domains were selection bias (allocation concealment) and selection of the reported result (result analyzed in accordance with a prespecified analysis plan); only 8 studies reached both criteria. A detailed risk-of-bias assessment for HAMD outcomes is presented in Figure 2.

### Effects of interventions

Table 1 summarizes the treatment effects, quality of evidence, and GRADE assessment of depression scores across all included trials.

**Table 1.** GRADE Summary of HAMD-17 for all Comparisons Among Trials Included

Comparisons	Studies	Effect estimates (95% CI)	I^2^ (%)	Quality of evidence
Acupuncture vs. blank control	Ai et al. 2018 Duan et al. 2011	−0.61 (−0.79, −0.43)	73.6	⊕ΟΟΟ Very Low
	Dai et al. 2024 Dilinuer et al. 2024			
	Fu et al. 2024 Gao et al. 2023			
	Hu et al. 2024 Jiang et al. 2012 Liu et al. 2015			
	Liu et al. 2015 Li et al. 2020 MacPherson et al. 2013 Tang et al. 2020 Wang et al. 2016 Wang et al. 2022 Wu et al. 2025 Wong et al. 2021 Xu et al. 2011 Zhu et al. 2018 Zhao et al. 2019 Zhao et al. 2024			
Acupuncture vs. SA/PA	Andreescu 2011 Ma et al. 2014	−1.12 (−1.57, −0.67)	88.2	⊕⊕ΟΟ Very Low
	Ma et al. 2020 Wang et al. 2017 Wu et al. 2025 Yindee et al. 2024 YinZ et al. 2022 Wong et al. 2021 Wang et al. 2021 Yin et al. 2020 Yeung et al. 2011 Quah-Smith et al. 2013 Song et al. 2009			
Acupuncture vs. antidepressant	Hu et al. 2024 Qu et al. 2013	−0.84 (−1.98, 0.29)	94.7	⊕ΟΟΟ Very Low
	Song et al. 2009 Wang et al. 2013			
	Wang et al. 2014			
EA vs. MA	Han 2019 Qu 2013 Xu 2011 Zhao 2019 Zhao 2024	−0.24 (−0.42, 0.07)	0.0	⊕⊕ΟΟ Low

Abbreviations: CI, confidence interval; EA, electroacupuncture; GRADE, Grading of Recommendations, Assessment, Development, and Evaluation; MA, manual acupuncture; PA, placebo acupuncture; SA, sham acupuncture; vs., versus.

### Depression score

**Figure 3.**
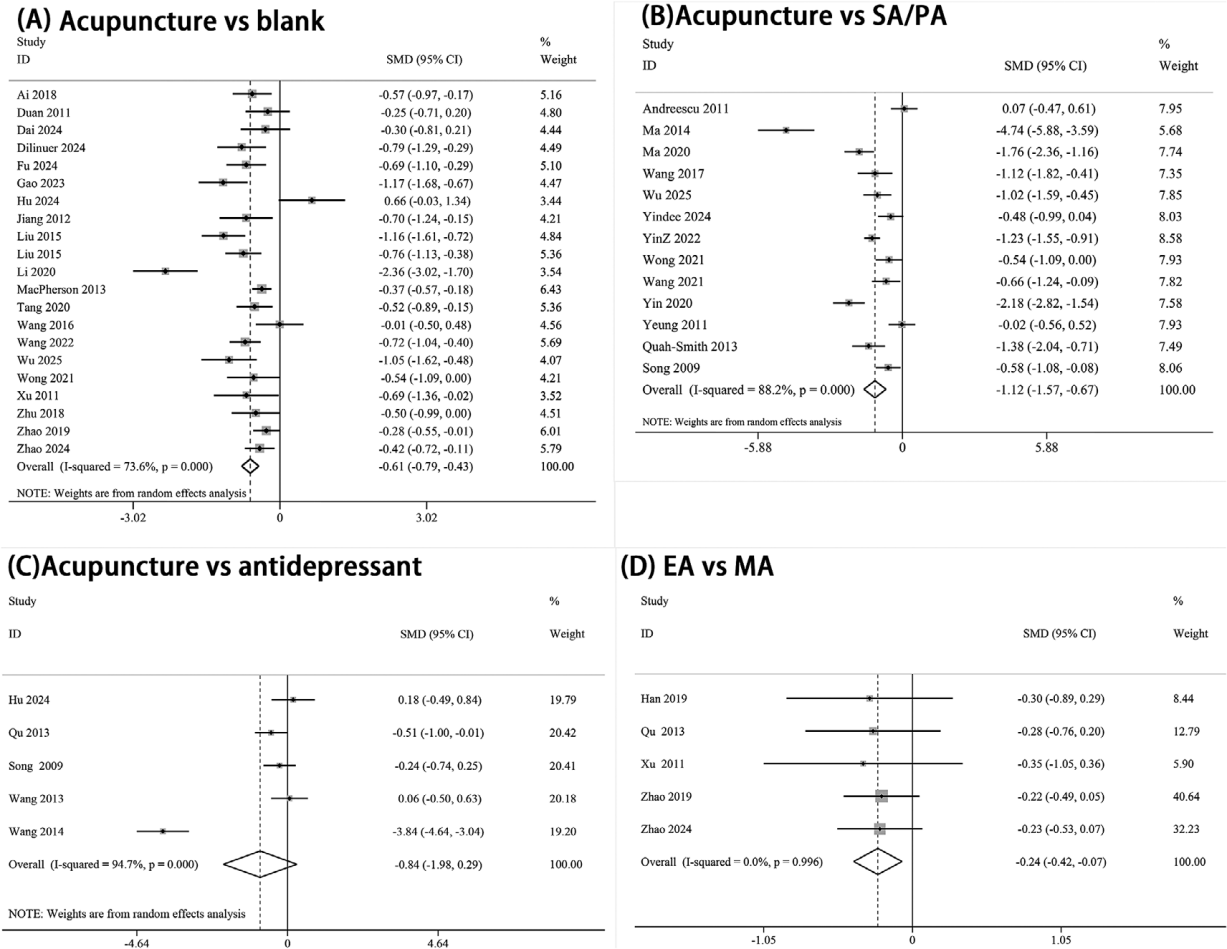
Forest plot for the reduction of depression scores of pre- and post-treatment change values. (A) Acupuncture vs. blank; (B) Acupuncture vs. SA/PA; (C) Acupuncture vs. antidepressant; (D) EA vs. MA. EA, electroacupuncture; MA, manual acupuncture; PA, placebo acupuncture; SA, sham acupuncture; vs., versus.

Very low-quality evidence suggests that acupuncture demonstrates superior antidepressant effects compared to no-treatment controls (SMD −0.61, 95% CI −0.79 to −0.43, *p* < 0.01). When compared to SA/PA, acupuncture demonstrated significantly greater antidepressant efficacy (SMD −1.12, 95% CI −1.57 to −0.67, *p* < 0.01). Very low-quality evidence indicates that the antidepressant effect of acupuncture is comparable to antidepressants (SMD −0.84, 95% CI −1.98 to 0.29, *p* > 0.05). Subgroup analysis revealed that EA may have better antidepressant effects than MA (SMD −0.24, 95% CI −0.42 to −0.07, *p* < 0.01). The results are illustrated in Figure 3.

### Adverse reactions

Twenty-one studies reported post-treatment safety outcomes, with 4 studies documenting no AEs across all treatment groups during the trial period and 2 studies reporting serious AEs. AEs were assessed using the UKU Side Effect Rating Scale in one study, the SERS in 5 studies, and the TESS in 2 studies. The most frequent acupuncture-related AEs were subcutaneous hemorrhage and hand numbness, while antidepressants were predominantly associated with gastrointestinal symptoms, dizziness, and somnolence. Meta-analysis indicated that acupuncture was associated with higher safety compared to control groups (RR: 0.53; 95% CI 0.30 to 0.96, *p* < 0.05).

### Acupuncture dose

**Figure 4.**
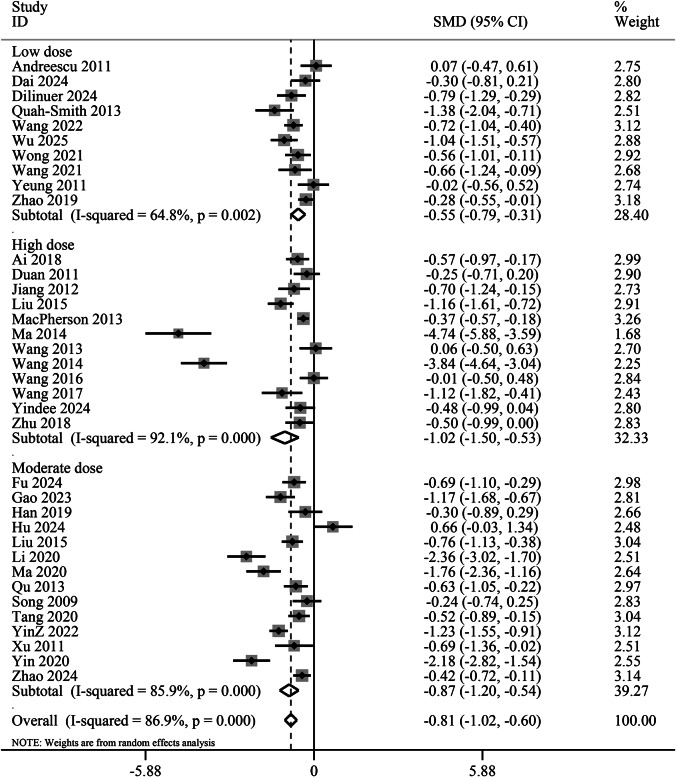
Subgroup analysis of different acupuncture doses for the reduction of depression scores. CI, confidence interval; SMD, standardized mean difference.

The total acupuncture dosage across included trials was categorized as high dose (33% of 36 trials), medium dose (39%), and low dose (28%). Subgroup analysis revealed that both high-dose (SMD −1.02, 95% CI −1.50 to −0.53, *p* < 0.01) and moderate-dose acupuncture (SMD −0.87, 95% CI −1.20 to −0.54, *p* < 0.01) demonstrated significantly greater antidepressant effects compared to low-dose treatment (SMD −0.55, 95% CI −0.79 to −0.31, *p* < 0.01) (Figure 4).

**Figure 5.**
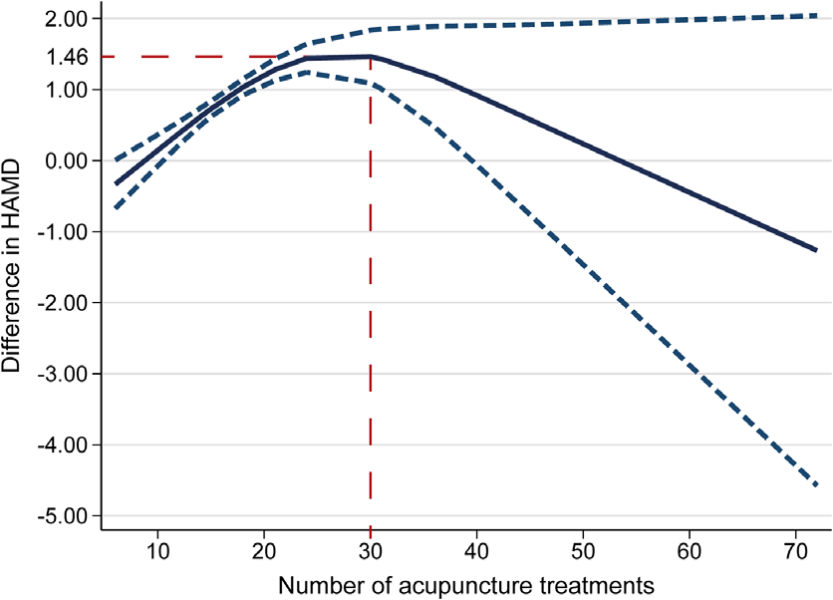
Dose–effect relationship between the number of acupuncture treatments and improvement of HAMD score. HAMD, Hamilton Depression Rating Scale.

We further explored the dose–effect relationship of acupuncture using the REMR method. Thirty-three clinical trials involving 37 acupuncture-related experimental groups were included for the dose–effect meta-analysis. Figure 5 shows the dose–effect relationship between the number of acupuncture sessions and the improvement of HAMD scores. As the number of acupuncture sessions increased from 12 times (0.39, 95% CI 0.22 to 0.55) to 30 times (1.46, 95% CI 1.09 to 1.84), the HAMD score significantly decreased, reaching a peak at 30 sessions. After 30 acupuncture sessions, the improvement rate gradually decreased (Figure 5).

### Course

**Figure 6.**
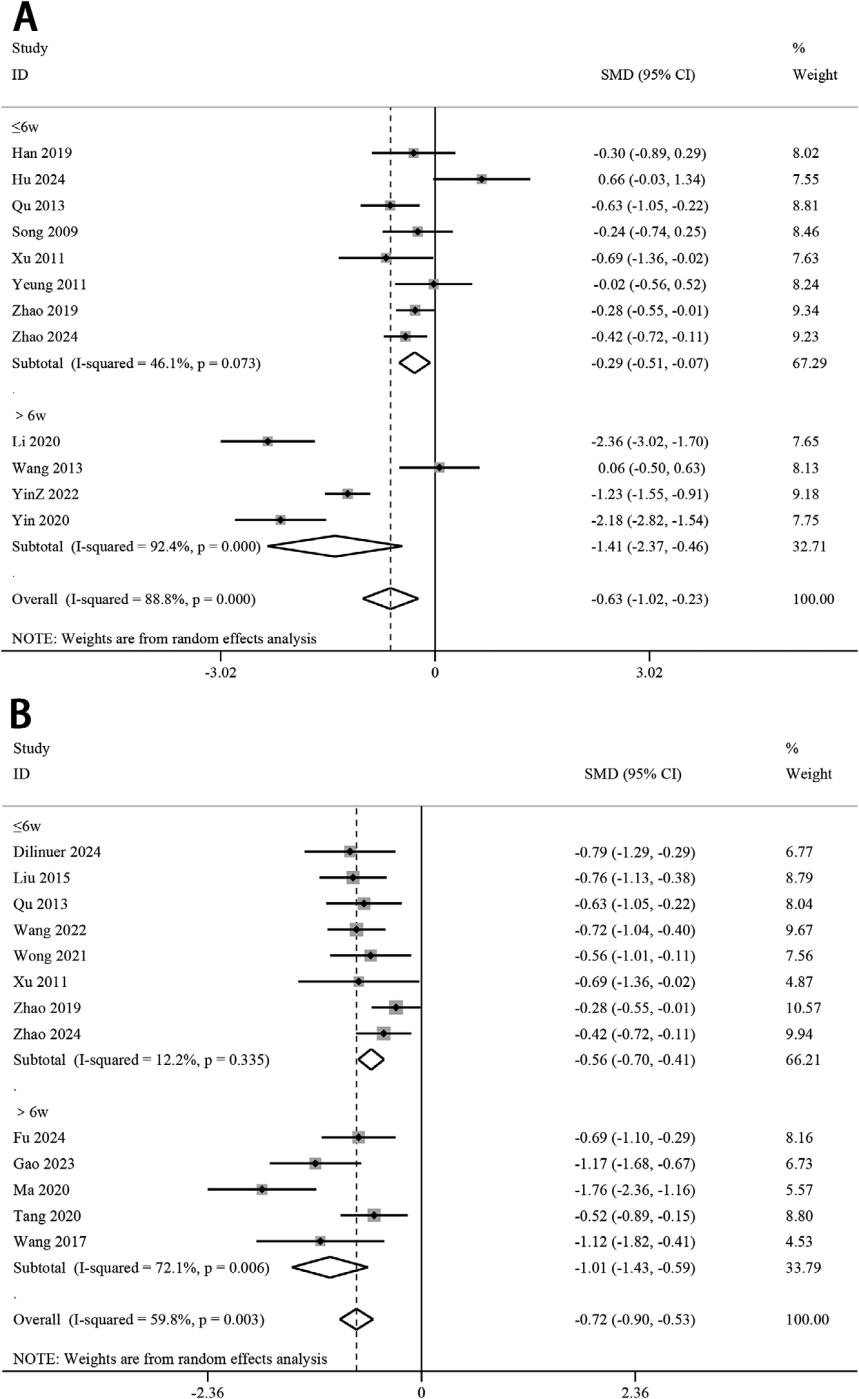
Meta-analysis on the course–effect of electroacupuncture (A) and manual acupuncture (B) on major depressive disorder. CI, confidence interval; SMD, standardized mean difference.

In EA, treatment courses exceeding 6 wk (SMD −1.41, 95% CI −2.37 to −0.46, *p* < 0.01) demonstrated superior antidepressant efficacy compared to those ≤6 wk (SMD −0.29, 95% CI −0.51 to −0.07, *p* < 0.05). Similarly, MA also showed significantly greater antidepressant effects when applied for >6 wk (SMD −1.01, 95% CI −1.43 to −0.59, *p* < 0.01) versus ≤6 wk (SMD −0.56, 95% CI −0.70 to −0.41, *p* < 0.01) (Figure 6).

### Frequency

**Figure 7.**
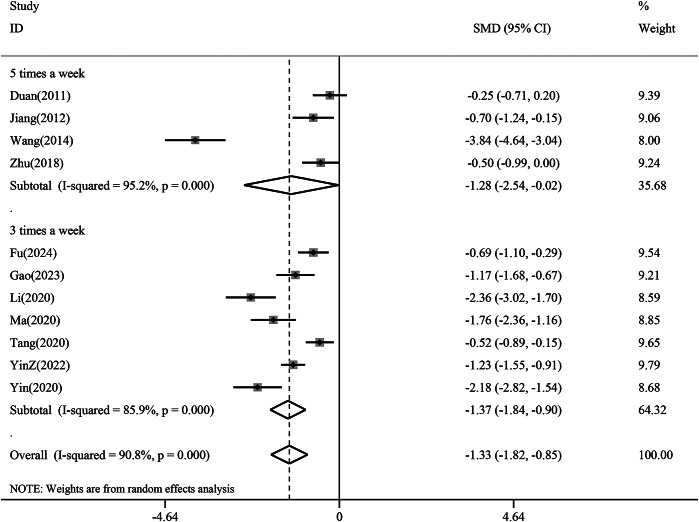
Subgroup analysis of treatment frequency for the reduction of depression scores. CI, confidence interval; SMD, standardized mean difference.

The subgroup analysis revealed a greater reduction in depression scores with treatment frequency 3 times a week (SMD −1.37, 95% CI −1.84 to −0.90, *p* < 0.01) than with treatment frequency 5 times a week (SMD −1.28, 95% CI −2.54 to −0.02,*p* < 0.05). This difference suggests that 3 times a week may be an appropriate treatment frequency in acupuncture for depression (Figure 7).

### Needling manipulation and retention time

**Figure 8.**
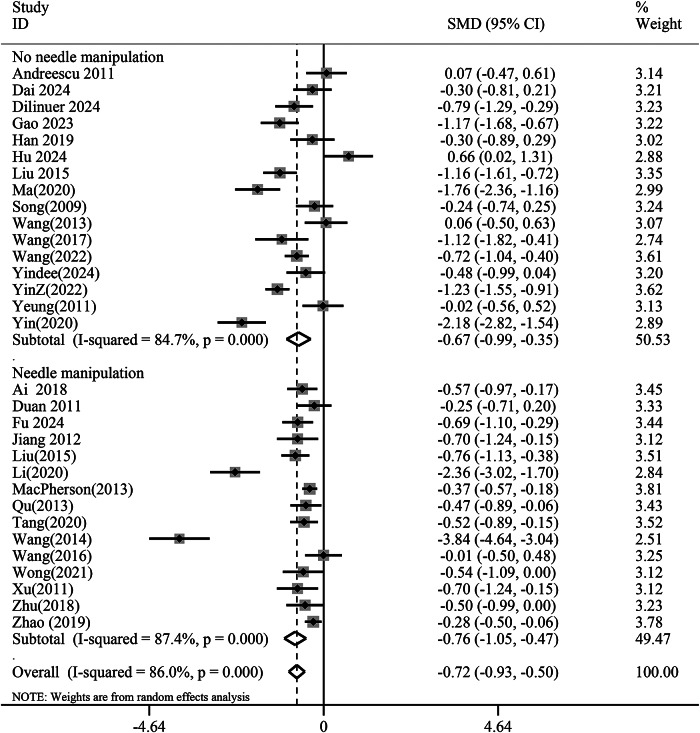
Subgroup analysis of needle manipulation versus no needle manipulation groups for the reduction of depression scores. CI, confidence interval; SMD, standardized mean difference.

**Figure 9.**
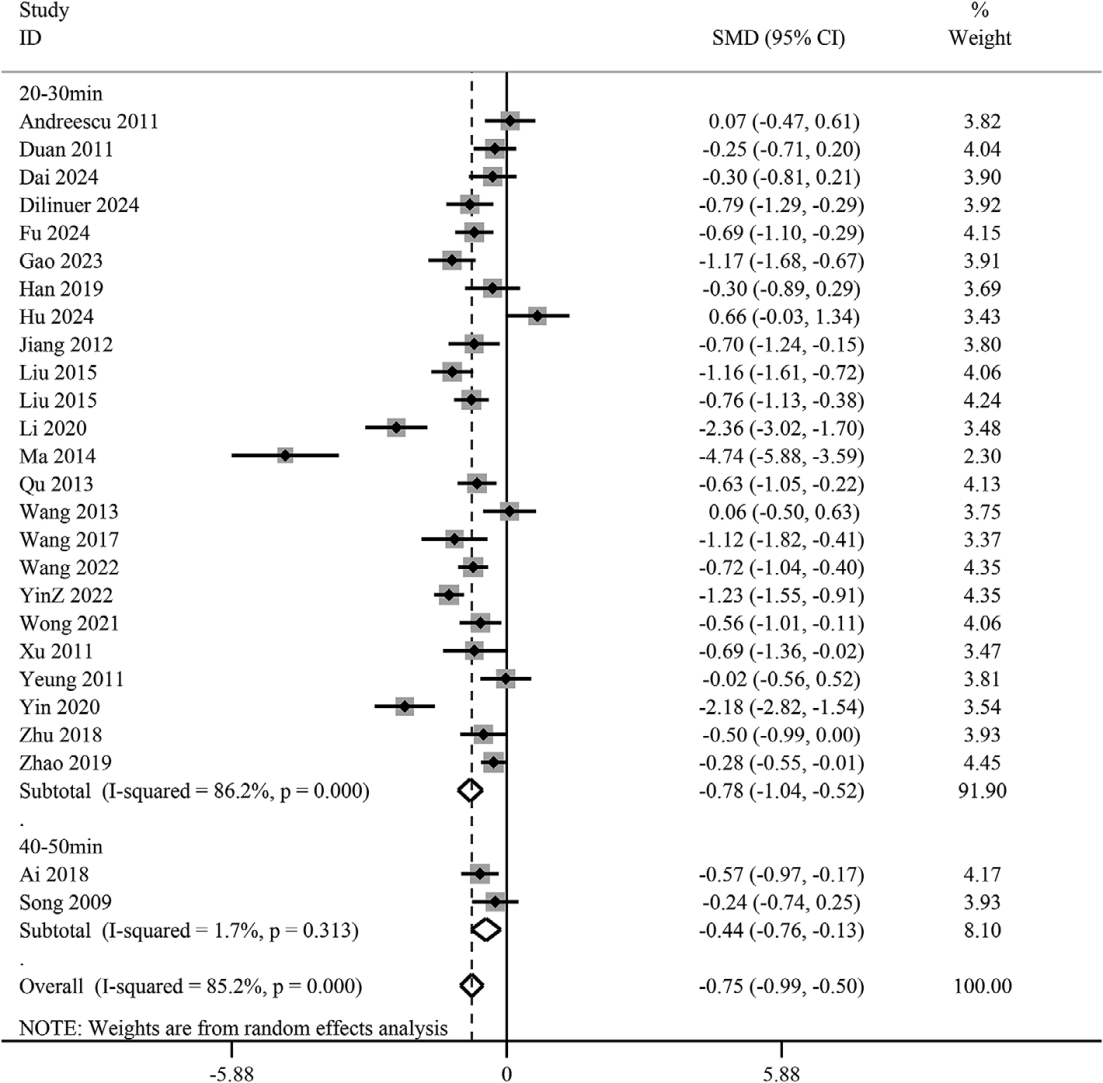
Subgroup analysis of needle retention time. for depression score reduction. SMD: standardized mean difference; CI: confidence interval.

Subgroup analysis indicated that the needling manipulation group (SMD −0.76, 95% CI −1.05 to −0.47, *p* < 0.01) did not demonstrate superior antidepressant effects compared to the non-needling manipulation group (SMD −0.67, 95% CI −0.99 to −0.35, *p* < 0.01), suggesting that needling techniques may not significantly influence acupuncture’s antidepressant efficacy (Figure 8). Furthermore, based on needle retention time, a duration of 20–30 min (SMD −0.78, 95% CI −1.04 to −0.52,*p* < 0.01) showed better antidepressant outcomes than 40–50 min (SMD −0.44, 95% CI −0.76 to −0.13, *p* < 0.01) (Figure 9).

**Figure 10.**
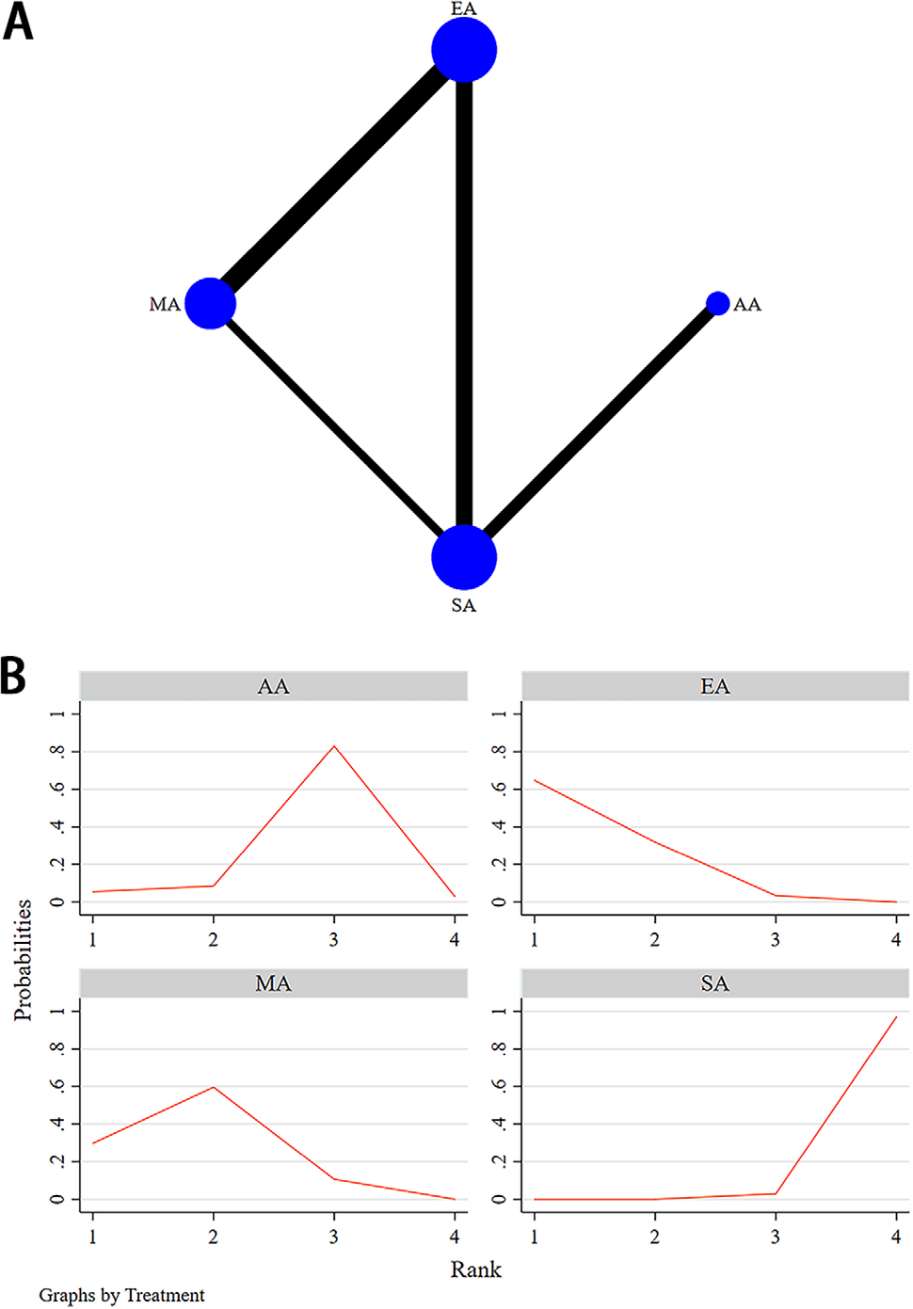
(A) Structure of network formed by interventions for depression relief. The size of the nodes represents the sample size of each intervention, and the thickness of the continuous lines connecting the nodes indicates the number of studies directly comparing the 2 interventions. (B) The SUCRA analysis and league table showed. AA, auricular acupuncture; EA, electroacupuncture; MA, manual acupuncture; PA, placebo acupuncture; SA, sham acupuncture.

### Acupuncture type

Fourteen RCTs involving 4 interventions—EA, MA, AA, and SA—examined their antidepressant efficacy for MDD patients; the network plots are presented in Figure 10A. The inconsistency assessment between direct and indirect comparisons based on the node-splitting model indicates no inconsistencies among all studies (*p* > 0.05); therefore, a consistency model was used. Detailed test results are shown in Table 2.

**Table 2.** Node-Splitting Analysis of Inconsistency

Comparison	Direct Coef.	Std. Err.	Indirect Coef.	Std. Err.	Difference Coef.	Std. Err.	*p-*value
AA vs. SA	3.89	1.82	5.48	557.98	−1.59	557.98	0.998
EA vs. MA	1.15	1.45	−2.16	3.06	3.31	3.38	0.33
EA vs. SA	6.55	1.76	9.87	2.89	−3.31	3.38	0.33
MA vs. SA	8.71	2.50	5.40	2.28	3.31	3.38	0.33

Abbreviations: AA, auricular acupuncture; Coef., coefficient; EA, electroacupuncture; MA, manual acupuncture; SA, sham acupuncture; Std. Err., standard error; vs., versus.

The NMA results did not further support the hypothesis that MA (SMD −4.82, 95% CI −10.87 to 1.23), EA (SMD −3.31, 95% CI −9.94 to 3.32), and AA (SMD −2.66, 95% CI −7.63 to 2.30) were more effective than SA in improving depressive symptoms. However, the NMA results showed that EA seemed to achieve superior outcomes compared to MA (SMD −8.71, 95% CI −13.63 to −3.82) (Figure 10A). The results of the NMA of different interventions were displayed in Supplementary File 4. The cumulative ranking plot indicated that EA had the highest probability of being the most effective intervention, followed by MA in second place, AA in third, and SA ranking last (Figure 10B).

## Discussion

### Main findings

To our knowledge, this study represents the most up-to-date and comprehensive systematic review with pairwise and exploratory NMAs on acupuncture for MDD. We evaluated the efficacy and safety of acupuncture in patients with MDD and also demonstrated that acupuncture significantly alleviates depressive symptoms compared to sham/placebo acupuncture, as supported by subgroup analyses. Furthermore, we found that the antidepressant effect of acupuncture was comparable to that of conventional antidepressant medications. However, unlike the common side effects of antidepressant medications such as dizziness, nausea, vomiting, fatigue, and later-stage issues like drug resistance and withdrawal reactions, acupuncture-related AEs—such as transient and mild subcutaneous bleeding, hand numbness, or brief dizziness—typically resolve after discontinuing treatment or with routine care. As a complementary and alternative therapy with minimal toxicity and favorable long-term efficacy, acupuncture has been recommended by multiple clinical guidelines as a non-pharmacological intervention for MDD.^27^^–^^29^

This systematic review critically examines the dose–effect, course–effect, frequency–effect, and acupuncture modalities–effect of acupuncture in MDD. Through subgroup analyses of acupuncture dosage, treatment course, needle retention time, treatment frequency, and needle manipulation, we demonstrated that moderate-to-high-dose regimens, treatment courses exceeding 6 wk, treatment frequency 3 times a week, and needle retention durations of 20–30 min had a better point estimate on depression relief. However, we could not perfectly address the issues related to statistical heterogeneity. Subsequently, we further quantified the dose–effect relationship of acupuncture for antidepressant effects through REMR analysis. The data revealed that the optimal therapeutic dose was achieved at 30 treatment sessions.

In the included 3-arm RCTs, direct comparisons between EA and MA for antidepressant efficacy did not reach statistical significance. However, subgroup analyses within the present study revealed superior therapeutic effects of EA over MA. This finding was further corroborated by NMA, which demonstrated that EA exhibited the strongest antidepressant effects among all acupuncture modalities (including MA and AA), but the outcome was associated with extremely high uncertainty.

### Comparison with existing studies

Two previous NMAs have examined the efficacy differences among various acupuncture modalities for MDD.^14^^,^^19^ To be specific, one NMA^19^ incorporating 71 RCTs confirmed that both EA and MA combined with SSRIs achieved favorable therapeutic outcomes, with EA exhibiting superior short-term efficacy compared to MA, which is partially consistent with the conclusions of our study. Another NMA^14^ encompassing 22 studies found that when combined with antidepressants, EA did not show significant benefits over SA. However, when compared to a waitlist control, EA combined with antidepressants emerged as an effective intervention. Our study similarly supports that acupuncture efficacy varies depending on the control group used, highlighting the critical importance of rigorous control group matching in clinical research to minimize bias. A recent meta-analysis^30^ further evaluated the efficacy differences among various acupuncture modalities combined with antidepressants through subgroup analysis, indicating that only MA combined with medication demonstrated significant antidepressant effects, while laser acupuncture and EA did not show obvious effects. This finding substantially diverges from our results, which may be attributed to various factors, including inconsistent inclusion/exclusion criteria, variations in acupuncture protocols and stimulation parameters, differences in MDD clinical subtypes and severity distributions, as well as inevitable regional bias.

This study represents the first pairwise meta-analysis on acupuncture for MDD, incorporating multiple control groups such as SA, placebo acupuncture, waitlist, and antidepressants. Current evidence from RCTs points to a significant dose–response relationship between the number of acupuncture sessions and clinical efficacy.^31^^,^^32^ Similar dose–effect relationships have also been validated in meta-analyses of chronic prostatitis/chronic pelvic pain syndrome and primary insomnia.^33^^,^^34^ The dose–effect model established in this study confirms that clinical effectiveness increased substantially until 30 acupuncture sessions were reached. This finding is highly consistent with the optimal therapeutic dose (36 sessions) proposed in previous meta-regression analyses.^18^

Our study included treatment courses ranging from 2 to 24 wk across the analyzed trials. Differing from the 4-wk treatment period suggested by previous meta-analyses,^15^ our finding indicates that a minimum of 6 wk for acupuncture intervention is required to demonstrate its antidepressant advantages. Given the progressive and cumulative nature of acupuncture’s regulatory mechanisms, a 6-wk intervention appears to be an appropriate treatment period.

### Strengths and limitations

Our meta-analysis possesses several notable strengths. First, this study represents the first application of pairwise meta-analysis in the field of acupuncture for MDD, systematically evaluating the dose–effect and acupuncture modalities–effect relationship through REMR and NMA, thereby providing higher-quality evidence for optimizing acupuncture treatment protocols. Second, our subgroup analysis clearly demonstrates that moderate-to-high-dose acupuncture is significantly more effective than low-dose interventions in alleviating depressive symptoms. By further employing the REMR model, we quantified the linear dose–effect relationship, identifying both the threshold dose for clinical effect and the optimal therapeutic dose. The determination of these critical dosimetric parameters not only facilitates protocol optimization to maximize clinical efficacy but also holds profound implications for advancing the standardization and scientific validation of acupuncture therapy. Third, we integrated both direct and indirect comparative evidence to evaluate the relative efficacy of EA, MA, and AA in improving depressive symptoms. An exploratory NMA further substantiated EA’s superior therapeutic advantage for MDD, despite the limited quality of evidence. Finally, through additional subgroup analyses, we investigated potential factors influencing acupuncture efficacy, including treatment courses, needle retention time, and needle manipulation techniques.

Our meta-analysis has several limitations. First, the issues of high risk of bias and low evidence quality remain unresolved. Despite rigorous literature screening and the exclusion of studies lacking reported randomization methods, ethical approval, or clinical trial registration, the overall evidence quality of the included studies was still relatively low, with significant bias present. In fact, we also conducted subgroup analyses to explore sources of heterogeneity, but heterogeneity persisted. Second, variations in other acupuncture parameters (eg, intervention duration, needle retention time, needle type, insertion direction, and depth), as well as differences in patient baseline characteristics, depression severity, gender distribution, and illness duration, made cross-study comparisons challenging. Individual sensitivity to acupuncture and potential rating scale errors may have further contributed to high heterogeneity. Furthermore, for acupuncture dosage assessment, we merely calculated total session numbers by multiplying treatment frequency and duration, while other critical factors—such as acupoint combinations, deqi response, and reinforcement/reduction techniques—lacked standardized quantification. Additionally, the number of studies included in certain intervals (eg, needle retention time of 40–50 min) was limited, which may affect the reliability of the specific findings for those subgroups. Finally, as MDD is a chronic neuro-inflammatory disorder with emotional dysregulation, the absence of follow-up data in this study precluded evaluation of acupuncture’s long-term effects, recurrence rates, and relapse prevention potential.

### Implications

Acupuncture’s treatment effect varies largely across trials, primarily attributable to the lack of standardized parameters in clinical guidelines—particularly regarding acupuncture modalities, treatment dosage, frequency, and course. As demonstrated in our study, EA exhibits superior effect sizes compared to traditional MA, owing to its synergistic combination of needling and low-frequency electrical stimulation that delivers more precise and controllable neuromodulatory “dosage.” Clinical evidence indicates that EA’s antidepressant efficacy parallels that of standard-dose fluoxetine.^35^ However, unlike the delayed onset of SSRIs (typically 2–4 wk),^36^ EA produces rapid therapeutic effects mediated by acute mobilization of monoaminergic systems and immediate activation of key brain regions, including the dorsal raphe nucleus, locus coeruleus, and ventral tegmental area.^37^^–^^39^ Extensive preclinical studies have validated EA’s sustained antidepressant effects, with mechanisms involving long-term potentiation in the ventromedial prefrontal cortex,^40^ modulation of the infralimbic-basolateral amygdala emotional circuit,^41^ persistent activation of monoaminergic neurotransmitter systems,^42^ and enhanced synaptic plasticity in hippocampal CA1 neurons.^43^ Among these, synaptic plasticity plays a pivotal role in MDD pathophysiology. EA achieves synaptic transmission and structural remodeling in the hippocampus by enhancing excitatory neurotransmitter transmission, activating plasticity-related signaling pathways such as BDNF/TrkB, cAMP/PKA/CREB, mTOR, and promoting the expression of synaptic-related proteins.^44^^–^^46^ Emerging evidence also identifies astrocytes as cellular targets of EA,^47^ with its antidepressant effects potentially mediated through reversal of astrocytic morphological atrophy and functional deficits, upregulating glutamate reuptake mediated by GLT-1 and BDNF secretion, thereby improving synaptic plasticity.

Acupuncture treatment exhibits a distinct dose-dependent effect profile. Unlike its rapid analgesic effects—mediated through immediate neural transduction pathways (where pain relief thresholds can be reached with as few as 6 sessions)—the antidepressant mechanisms of acupuncture involve more complex neuro-endocrine-immune network modulation. This requires a greater number of interventions to cross the clinical threshold and achieve a qualitative therapeutic shift.^48^ Further studies reveal that MDD’s dose–effect relationship depends on the cumulative effects of neural plasticity.^49^^,^^50^ EA-induced synaptic changes associated with depression alleviation are progressively amplified with repeated treatments, ultimately resulting in the observed cumulative antidepressant effects. Specifically, a certain amount of acupuncture treatment is required to cross the neuromodulatory dose window and trigger synaptic plasticity remodeling. Thirty sessions typically yield peak antidepressant efficacy, marked by a plateau in neural plasticity biomarker expression and stabilization of functional connectivity, followed by a saturation phase accompanied by diminishing marginal effects.

In the calculation of acupuncture dosage, treatment frequency serves as a critical variable. Internationally, most therapeutic protocols adopt a regimen of 1–2 sessions/wk,^51^ though such prolonged inter-stimulus intervals may lead to suboptimal cumulative effects. By contrast, a higher frequency of 3 sessions/wk is more commonly employed in clinical practice within China, which is consistent with our findings. Evidence-based clinical experience suggests that a stepped treatment approach—beginning with an initial high-frequency phase (over 3 sessions/wk) for intensive therapy, followed by a low-frequency maintenance phase (1–2 sessions/wk)—may represent an optimized strategy.^52^^,^^53^ Currently, there is limited literature investigating dose–effect and treatment course–effect relationships, with most studies focusing on isolated variables rather than developing integrated multiparameter models or dynamic monitoring frameworks. Future research should emphasize comprehensive multifactorial analysis to establish more precise and personalized clinical guidance protocols.

## Conclusions

Current low-quality evidence suggests that acupuncture, particularly electroacupuncture, may be an effective intervention for MDD, with its clinical efficacy potentially influenced by treatment dosage parameters, therapeutic course, treatment frequency, and needle retention time. Electroacupuncture, higher-dose acupuncture, treatment courses exceeding 6 wk, treatment frequency 3 times a week, and needle retention durations of 20–30 min may constitute critical determinants of therapeutic effectiveness. However, the evidence is highly uncertain due to its low quality. Future research employing rigorous study designs and higher evidence standards is urgently needed to (1) validate its therapeutic equivalence with pharmacotherapy and (2) establish optimal treatment parameters.

## Supporting information

10.1017/S1092852926100868.sm001 Zhao et al. supplementary material Zhao et al. supplementary material

## Supplementary material

The supplementary material for this article can be found at http://doi.org/10.1017/S1092852926100868.
