# Supplementary material for: Efficacy and influencing factors of acupuncture in major depressive disorder: a systematic review and exploratory network meta-analysis
Source: CNS Spectr. 2026 Mar 2;31(1):e6. doi: 10.1017/S1092852926100868 (PMC13076089; doi:10.1017/S1092852926100868)
Supplement: Zhao et al. supplementary material [file S1092852926100868sup001.docx]

**Title:** Efficacy and Influencing Factors of Acupuncture in Major Depressive Disorder: A Systematic Review and Exploratory Network Meta-Analysis

**Supplemental file 1. Search Strategy**

**1. PubMed**#1 “Acupuncture” [Mesh]

#2 “Acupuncture Therapy” [Mesh]

#3 “Electroacupuncture” [Mesh]

#4 Acupunctur* [tiab]

#5 Acupressure [tiab]

#6 Electroacupuncture [tiab]

#7 electrostimulation [tiab]

#8 auriculoacupuncture[tiab]

#9 OR #1-8

#10 “Depressive Disorder, Major” [MeSH]

#11 “Depression” [MeSH]

#12 “Depressive Disorder” [MeSH]

#13 Depression [tiab] OR Depressive Disorder [tiab] OR Depressive Disorder, Major [tiab] OR Depressive Symptom*[tiab] OR Symptom, Depressive[tiab] OR Emotional Depression[tiab] OR Depression, Emotional[tiab] OR Depressive Disorders, Major[tiab] OR Major Depressive Disorder*[tiab] OR Clinical Depression[tiab] OR Depression, Involutional[tiab] OR Involutional Depression[tiab] OR Melancholia, Involutional[tiab] OR Involutional Melancholia[tiab] OR Psychos*, Involutional[tiab] OR Involutional Psychos*[tiab] OR Paraphrenia*, Involutional[tiab] OR Involutional Paraphrenia*[tiab]

#14 OR #10-13

#15 “Randomized Controlled Trial” [Publication Type] OR “Randomized Controlled Trials as Topic” [MeSH]

#16 #9 AND #14 AND #15

**2. Web of science**

#1 TS=(Depression OR Depressive Disorder OR Major Depressive Disorder* OR Clinical Depression OR Depressive Symptom* OR Emotional Depression OR Clinical Depression OR Involutional Depression OR Involutional Melancholia OR Involutional Psychos* OR Involutional Paraphrenia*)

#2 TS=(acupuncture OR acupuncture therapy OR electroacupuncture OR acupunctur* OR acupoint* OR acupotom* OR Pharmacopuncture OR needle OR electrostimulation OR auriculoacupuncture)

#3 TS=(Randomized Controlled Trial OR Randomized Controlled Trials as Topic Single-Blind Method OR Double-Blind Method OR Random Allocation OR placebo)

#4 #3 AND #2 AND #1

**3. Embase**

#1 'depression'/exp OR 'depression' OR 'depressive disorder':ab,ti OR 'major depression,ti OR 'depressive symptom': ab,ti OR 'major depressive disorder ': ab,ti OR 'involutional depression':ab,ti OR 'involutional psychosis ': ab,ti OR 'emotional depression':ab,ti

#2 'acupuncture'/exp OR 'acupuncture' OR 'acupuncture therapy':ab,ti OR electroacupuncture:ab,ti OR 'acupuncturepoint':ab,ti OR 'acupotomy':ab,ti OR 'pharmacopuncture':ab,ti OR needle:ab,ti OR 'electrotherapy':ab,ti OR 'auricular acupuncture':ab,ti OR auriculotherapy:ab,ti

#3 'randomized controlled trial'/exp OR 'randomized controlled trial' OR 'randomized controlled trial ': ab,ti AND topic: ab, ti

#4 #1 AND #2 AND #3

**4. Cochrane library**

**#1** Depression [all text]

**#2** Depressive Disorder, Major [all text]

**#3** ("depression"): ti.ab.kw OR ("depressive disorder*"): ti.ab.kw OR ("major depressive disorder"): ti.ab.kw OR (Depressive Symptom*): ti.ab.kw OR (Emotional Depression): ti.ab.kw

**#4**  #1 OR #2 OR #3

**#5** Acupuncture [all text]

**#6** ("acupuncture"): ti.ab.kw OR (acupuncture therapy): ti.ab.kw OR ("acupoint*"): ti.ab.kw OR (electroacupuncture*): ti.ab.kw OR (auriculoacupuncture): ti.ab.kw

**#7** #5 OR #6

**#8** Randomized Controlled Trials as Topic [all text]

**#9** (Randomized Controlled Trials): ti.ab.kw OR (trial): ti.ab.kw

**#10** #8 OR #9

**#11** #4 AND #7 AND #10

**5. CNKI/ VIP/ Wan Fang/Sinomed**

The Chinese version available on request from reviewers or readers.

**Supplemental file 2. Funnel plot for Depression scores of acupuncture vs blank**

**
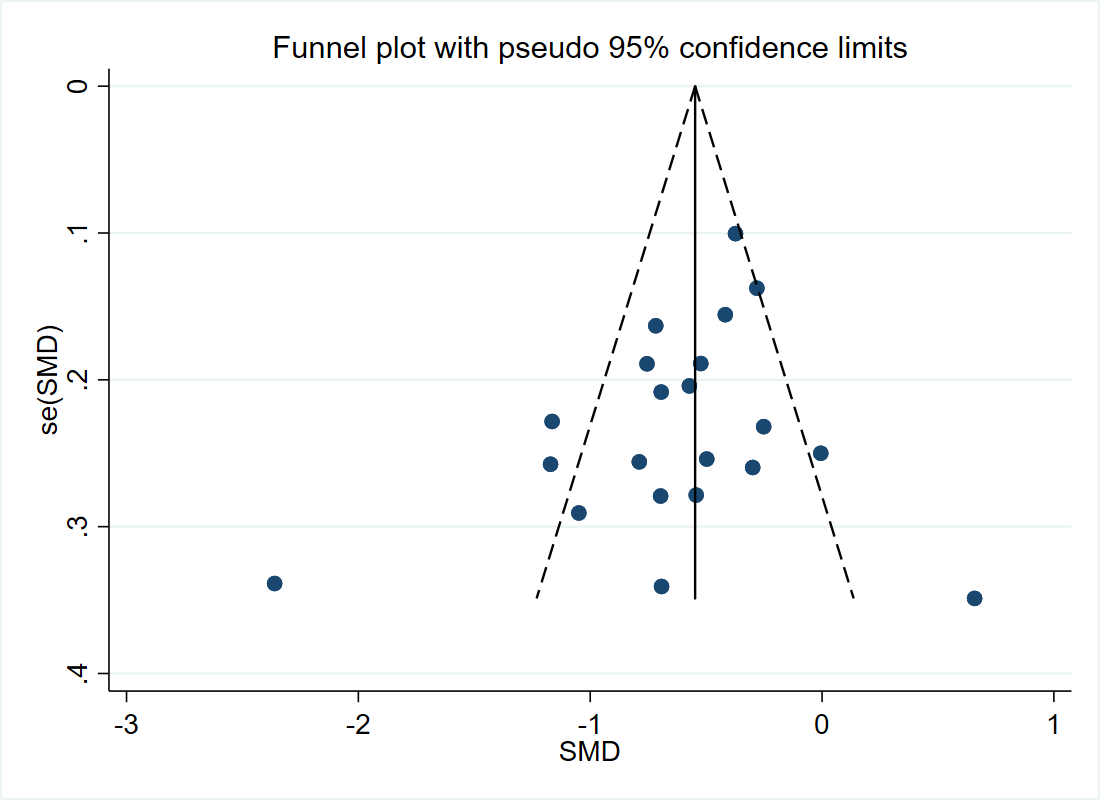
**

**Funnel plot for Depression scores of acupuncture vs SA /PA**

**
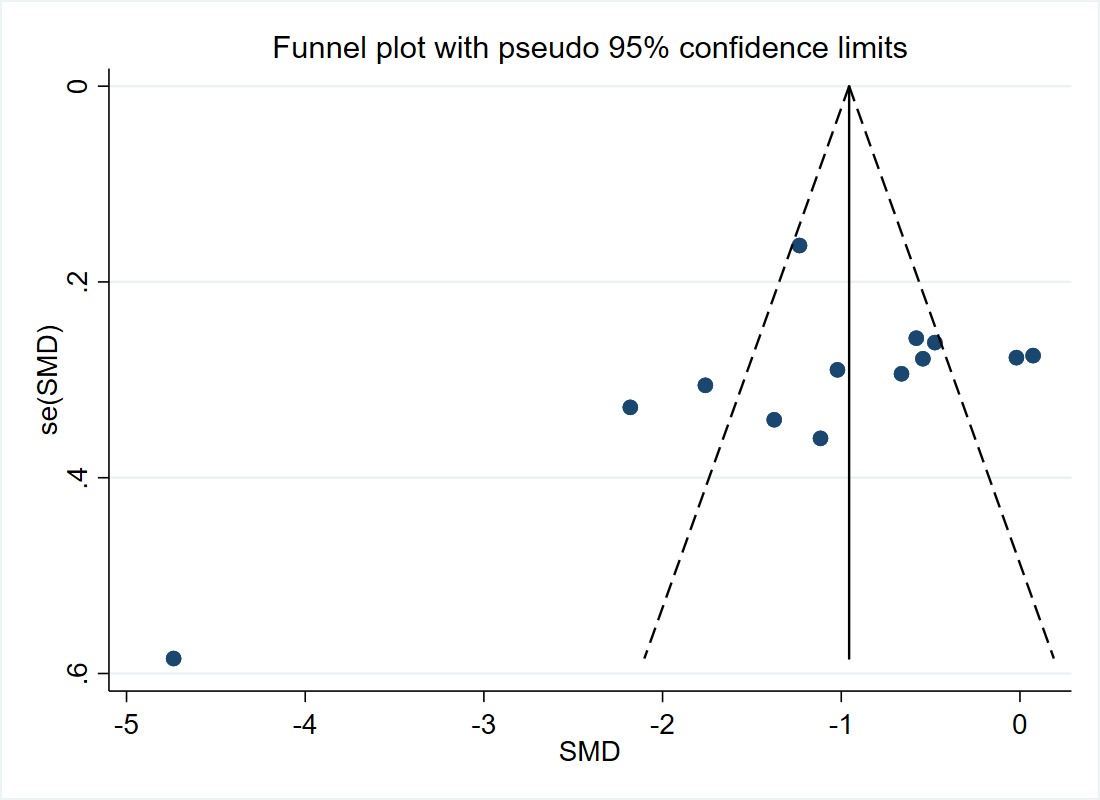
**

| **Supplemental file 3. Characteristics of the included studies(n=36)** | | | | | | | | | |
| --- | --- | --- | --- | --- | --- | --- | --- | --- | --- |
| **Author**  **(year)** | **Diagnostic criteria** | **Age** | **T**  **(sample)** | **C**  **(sample)** | **Intervention**  **Frequency and duration** | **Session** | **Course**  **(week)** | **outcome** | **AE** |
| Andreescu et al.  (2011) | DSM-IV | T:46.0±11.5  C:49.1±14.0 | EA  (28) | Sham EA  (25) | 2 times per week for 30 minutes for 6 weeks | 12 | 6 | HAMD | R |
| Ai et al.  (2018) | DSM-V | T:20.1±3.6  C:20.2±3.5 | MA  (50) | Blank  (50) | once a day for 50 min for 6 weeks | 42 | 6 | HAMD | R |
| Duan et al.  (2011) | ICD-10 | 35±8 | EA  (38) | Blank  (37) | 5 times per week for 30 min for 6 weeks | 30 | 6 | HAMD | NR |
| Dai et al.  (2024) | DSM | T: 30±8  C: 30±8 | EA  (30) | Blank  (30) | 5 times per week for 30 min for 3 weeks | 15 | 3 | HAMD | R |
| Dilinuer et al.  (2024) | ICD-10 | T:52.11±20.63  C:54.42±19.66 | MA  (33) | Blank  (33) | 3 times per week for 20 min for 4 weeks | 12 | 4 | HAMD | R |
| Fu et al.  (2024) | DSM-V | NR | MA  (51) | Blank  (47) | 3 times per week for 30 min for 8 weeks | 24 | 8 | HAMD | R |
| Gao et al.  (2023) | DSM-V | T:42±12  C:38±11 | MA  (39) | Blank  (39) | 3 times per week for 30 min for 8 weeks | 24 | 8 | HAMD | NR |
| Han et al.  (2019) | DSM-V | T: 36.8±7.47  C: 40.25±9.04 | EA  (23) | MA  (22) | 3 times per week for 30 min for 6 weeks | 18 | 6 | HAMD | NR |
| Hu et al.  (2024) | ICD-10 | T: 38.9± 12.96  C: 34.5±10.41 | EA  (20) | Blank  (16) | 3 times per week for 30 min for 6 weeks | 18 | 6 | HAMD | NR |
| Jiang et al.  (2012) | CCMD-3 | T: 37± 10  C: 36± 10 | MA  (25) | Blank  (30) | 5 times per week for 30 min for 6 weeks | 30 | 6 | HAMD | NR |
| Liu et al.  (2015) | CCMD-3 | T:36±11 C:37±11 | MA  (45) | Blank  (45) | Every other day for 30 min for 4 weeks | 14 | 4 | HAMD | NR |
| Liu et al.  (2015) | ICD-10 | T:37±10 C:36±10 | MA  (30) | Blank  (30) | every other day for 30 min for 6 consecutive weeks | 21 | 6 | MADRS | R |
| Li et al.  (2020) | DSM-IV | T:40.30±10.99 C:38.75±11.45 | EA  (30) | Blank  (30) | 3 times per week for 30 min for 8 weeks | 24 | 8 | HAMD | NR |
| MacPherson et al.  (2013) | NR | T:43.4±13.24 C:43.5±3.5 | MA  (302) | Blank  (151) | 12 sessions weekly for 3 months | 36 | 3 | PHQ-9 | R |
| Ma et al.  (2020) | CCMD-3  DSM-IV | ranging from 22 to 70 | MA  (30) | Sham MA  (30) | 24 consecutive treatments | 24 | 8 | HAMD | + |
| Qu et al.  (2013) | ICD-10 | MA:32.3±9.6 EA:33.2±9.0 C:34.4±10.8 | MA(51)  EA(49) | PRX  (43) | 3 sessions per week for 30 min for 6 weeks | 18 | 6 | HAMD | + |
| Quah-Smith et al.  (2013) | DSM-IV | T:40.08±9.37 C:36.27±10.13 | LA  (21) | PA  (22) | twice a week for 4 weeks and once a week for another four weeks, for a total of 12sessions. | 12 | 4 | HAMD | + |
| Song et al.  (2009) | DSM-IV | EA:30±11 fluoxetine:34±13  PA:32±12 | EA  (31) | fluoxetine(32) PA(32) | 3 times per week for 6 weeks | 18 | 6 | HAMD | NR |
| Tang et al.  (2020) | DSM-V | T:42±6 C:43±6 | MA  (58) | Blank  (58) | 3 times per week for 8 weeks | 24 | 8 | HAMD | + |
| Wang et al.  (2013) | DSM-IV | T:48.10±13.40 C:47.10±10.60 | EA  (24) | Paroxetine  (24) | 3 times per week for 20min for 24 weeks | 72 | 24 | MADRS  SDS | NR |
| Wang et al.  (2014) | ICD-9 | range 22 to 73 years | MA  (45) | Blank  (26) | 5 times per week for 6 weeks | 30 | 5 | HAMD | - |
| Wang et al.  (2016) | DSM-Ⅳ | T:41.3±5.2 C:42.1±4.7 | MA  (32) | Blank  (32) | 6 times per week for 8 weeks | 48 | 8 | HAMD | + |
| Wang et al.  (2017) | ICD-10 | T:44.5±10.47 C:43.78±9.10 | MA  (18) | SA  (18) | once a day for the first three days and subsequently once every three days for the remainder of the 8-week trial(20min) | 31 | 8 | MADRS  SDS | NR |
| Wang et al.  (2022) | DSM-IV | T:47.9±6.5 C:46.3±7.2 | MA  (80) | Blank  (80) | 5 times per week for 30min for 3 weeks | 15 | 3 | HAMD | + |
| Wu et al.  (2025) | ICD-10 | T:30.0±10.8  C:31.2±10.8 | AA  (40) | sham AA  (40) | total 10 times for 6 weeks | 10 | 6 | HAMD  SDS | + |
| Yindee et al.  (2024) | DSM-V | T:28.77±9.16  C:27.57±8.08 | EA  (30) | Sham EA  (30) | once a week for 20min for 10 weeks | 70 | 10 | PHQ-9 | NR |
| YinZ et al.  (2022) | DSM-V | T:50.9±14.0 C:50.5±14.0 | EA  (90) | Sham EA  (90) | 3 sessions per week for 30 min for 8 weeks | 24 | 8 | HAMD  SDS | + |
| Wong et al.  (2021) | DSM-V | T:44.8±10.3 C:50.9±11.1 | MA  (40) | Blank  (20) | 2 sessions per week for 30min for 3 weeks | 6 | 3 | HAMD  PHQ-9 | NR |
| Wang et al.  (2021) | DSM-IV | T:54.5±8.31 C:51.3±12.21 | AA  (25) | sham AA  (24) | 5 sessions per week for 30min for 2 weeks | 10 | 2 | HAMD  SDS | NR |
| Xu et al.  (2011) | CCMD-3 | MA:48.01±8.16 EA:47.54±8.03 C:47.42±8.89 | MA(25)  EA(20) | Blank  (30) | 3 sessions per week for 6 weeks | 18 | 3 | HAMD | NR |
| Yeung et al.  (2011) | DSM-IV | T:40.08±9.37 C:36.27±10.13 | EA  (26) | PA  (26) | 3 times per week for 3 weeks | 9 | 3 | HAMD | + |
| Yin et al.  (2020) | DSM-IV | T:47.30±14.89 C:46.77±15.57 | EA  (30) | Sham EA  (30) | 3 times weekly for 8 consecutive weeks | 24 | 8 | HAMD  SDS | + |
| Zhu et al.  (2018) | CCMD-3 | T:42.9±5.0 C:42.1±4.3 | MA  (32) | Blank  (32) | 1 session each Monday to Friday for a total of 30 sessions | 30 | 6 | HAMD | NR |
| Zhao et al.  (2019) | ICD-10 | MA:41.42±12.53 EA:41.18±12.00 C:41.76±12.85 | MA(161)  EA(160) | Blank  (156) | 3 sessions a week for 2 weeks | 6 | 2 | HAMD  SDS | + |
| Zhao et al.  (2024) | NR | T:41.39±12.58 C:40.75±12.21 | MA(128)  EA(123) | Blank  (123) | 3 sessions per week for 6 weeks | 18 | 6 | HAMD  SDS | + |

Abbreviations: T: treatment group; C: control group; R: reported; NR: unreported; HAMD: Hamilton Depression Scale; SDS: Self-Rating Depression Scale; PHQ-9: Patient Health Questionnaire; MADRS: Montgomery-Asberg Depression Rating Scale; MA: manual acupuncture; EA: electroacupuncture.

**Supplemental file 4.** **Results of network meta-analysis for all possible treatment effects.**

**
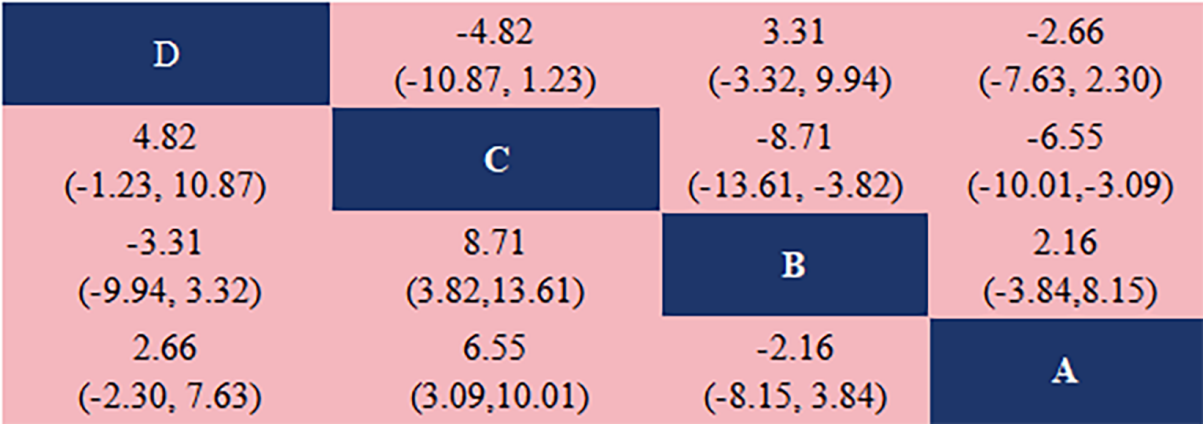
**
